# Investigation of the Quality of Life of Patients with Gastrointestinal Issues Treated in the Surgical Clinic of a Regional General Hospital in Greece

**DOI:** 10.3390/clinpract13020048

**Published:** 2023-04-12

**Authors:** Nikos Rikos, Chara Frantzeskaki, Maria Fragiadaki, Anna Kassotaki, Andreas Mpalaskas, Manolis Linardakis, Georgios Vasilopoulos

**Affiliations:** 1 Department of Nursing, School of Health Sciences, Hellenic Mediterranean University, 71410 Heraklion, Greece; 2 Venizelio General Hospital of Heraklion, 71410 Heraklion, Greece; 3 Department of Social Medicine, School of Medicine, University of Crete, 71410 Heraklion, Greece; 4 Department of Nursing, School of Health Sciences, University of West Attica, 12243 Athens, Greece

**Keywords:** gastrointestinal disorders, quality of life, psychological consequences, intestinal issues

## Abstract

As disorders of the gastrointestinal system are among the most prevalent, had a significant impact on patients’ quality of life (QoL). This study aimed to investigate the QoL of 83 patients treated in the surgical clinic of a regional general hospital in Crete-Greece. They recruited from April-to-June 2021 using the 36-Item Short Form Survey scale (SF-36) and the Spielberger State-Trait Anxiety Inventory (STAI). Logistic regression analysis was performed to evaluate the QoL in relation to patients’ characteristics. The 50.6% were men with mean age of all the 55.0 years. Mental Health was found with highest mean score in relation to Role Physical (*p* < 0.001) and the Mental Component higher than the Physical (*p* = 0.029) while 68.7% of patients had high State Anxiety score. For each added year of age, the odds ratio of a moderate-to-high Physical Component was significantly decreased (OR = 0.95, *p* = 0.012) as married/partnered patients had higher odds of a moderate-to-high Mental Component (OR = 6.59, *p* = 0.006). Those with high state anxiety had lower odds of a moderate-to-high Mental Component (OR = 0.16, *p* = 0.005). Action is necessary on a clinical or individual level, guided by health professionals, to promote appropriate dietary choices and interventions for the avoidance of behaviors that could harm or endanger patients’ health.

## 1. Introduction

The gastrointestinal (GI) system is responsible for the digestion and absorption of ingested food and liquids. GI diseases refer to diseases of the esophagus, stomach, small intestine, colon and rectum. Disorders of the GI system are among the most prevalent disorders, the main ones being esophageal and swallowing disorders, peptic ulcers, irritable bowel syndrome (IBS) and inflammatory bowel disease (IBD) [1].

Functional gastrointestinal disorders (FGIDS), which are the most common type, belong to a very widespread group of heterogenous disorders with increasing prevalence, difficulties in diagnosis, and low quality of life (QoL). These disorders affect up to 40% of people at any time in their lives. Psychological comorbidity and by extension the QoL of these patients is affected to such a degree that there is a significant impact both on the patients’ psychosocial status and on healthcare systems, meaning that early diagnosis is vitally important [2,3]. More specifically, FGIDs or disorders of gut-brain interaction (DGBIs) are GI disorders related to any combination of motility disturbance, visceral hypersensitivity, altered mucosal and immune function, altered gut microbiota, and altered central nervous system processing, resulting in unsuccessful stimulus processing [4]. They represent a significant economic burden on healthcare worldwide, and reduced quality of life associated with health [5,6]. The most common FGID is IBS, which affects the lower GI tract [7]. Psychiatric disorder co-morbidity is high in FGIDs patients, indicating common or interacting disease mechanisms, perhaps associated with communication between the gut and the brain, in which variations in the gut microbiome may play a key part [8,9]. A recent review notes that a high correlation is observed between psychiatric symptoms associated with stress, including anxiety, and IBS, providing a basis for further studies [8].

The main aim of the present study was to investigate the quality of life of patients suffering from disorders of the gastrointestinal tract who were treated in the surgical clinic of a regional general hospital in Greece. Subsidiary objectives were to measure factors affecting patients’ quality of life and their psychosocial status.

## 2. Materials and Methods

### 2.1. Study Design, Sample and Participants

The present study was a cross-sectional study using a questionnaire. The chosen sampling method was purposive sampling. The main exclusion criteria were language, not being an adult, and not being hospitalized for GIs. The final study sample consisted of 83 patients. Their main characteristics were: (a) they were over 18 years of age, (b) they were being treated in the surgical clinic for GI issues, (c) they had the mental capacity to understand the aim of the study and complete the questionnaire, and (d) they agreed to complete the questionnaire after reading and signing the printed consent form.

### 2.2. Research Tools

The research tools used were the 36-Item Short Form Survey (SF-36), which consists of the following sections: demographic data, characteristics of the disorder and habits, eating habits, perception of health and daily activities. The scale reliability of the SF-36 showed high consistency (Cronbach’s α = 0.941) with reversal of the responses to some of the 36 questions, divided into 8 physical and mental health components [10,11].

The second tool used was the Spielberger State-Trait Anxiety Inventory (STAI). This was used to investigate the patients’ psychosocial status. It consists of 40 statements which people often use to describe themselves. The first 20 items represent how they feel at this moment (state anxiety), while the second 20 assess how they feel in their life in general (trait anxiety) [12]. The cut-off of >40 was taken for the high anxiety prevalence [13]. Scale reliability was again obtained using Cronbach’s α = 0.807.

### 2.3. Data Collection

Data collection was carried out at the Surgical Clinic of the Venizelio-Pananeio General Hospital of Heraklion over the course of two months, from 24 April to 28 June 2021. The researcher met the participants inside the clinic. The purpose of the study was explained to each participant. After studying and stating that they understood the special form accompanying the questionnaires, participants gave their signed consent to continue the process. For the purposes of the study, participants had to answer all the questions on their medical history and quality of life, and a set of questions including demographic data such as age, gender, etc. Participants had the right to refuse to answer any question which might embarrass them or which they considered irrelevant to them. At no point was personal information, such as the patient’s name, requested, while the answers were completely confidential and used only for the purposes of the study.

### 2.4. Ethical Considerations

Ethical approval was obtained from the Research and Bioethics Committee (IRB; Hellenic Mediterranean University, Crete 495/53/08-03-2021) and the Venizelio General Hospital of Heraklion, Crete (43/5/22-04-2021). The participants in the study were informed about the study objectives, expected outcomes, and associated benefits and risks. Written consent was received from the participants before they answered the questionnaire. The authors also obtained permission to use the hospital facilities before data collection.

### 2.5. Statistical Analysis

Analysis was performed using SPSS (IBM Corp. Released 2019, IBM SPSS Statistics for Windows, v.26.0, Armonk, NY, USA: IBM Corp.). The frequency distributions of descriptive and other characteristics of the study participants were calculated. The shape of the distributions of the QoL and state anxiety scores was reviewed using Blom’s method (Q-Qplot) and their reliability factors were calculated using Cronbach’s alpha. A slight asymmetry was observed in components, which were compared using Kruskal-Wallis and Mann-Witney methods. Multiple logistic regression analysis was used to calculate the odds ratio (OR), correlating increased QoL in the Physical and Mental Components with the patients’ characteristics and their state anxiety. The significance level was set at 0.05.

## 3. Results

### 3.1. Patient Characteristics

Of the 83 patients who participated in the study (Table 1), the majority were men (50.6%) and the mean age of all was 55.0 ± 17.4 years (range 18 to 93 years), with 42.2% under the age of 50 years. 69.9% were married, 83.1% had children, 94.0% were Greek and 78.3% were urban residents. They were evenly distributed in all levels of education, while 42.1% had completed university or postgraduate education. The majority, 43.4%, reported a monthly income of 1000+ euros. The frequency distribution of the reasons for hospitalization of the 83 patients in the Surgical Clinic (results not shown in table) showed that the most frequent cause was “cholecystitis, cholelithiasis, cholangitis” (37.3%), followed by “acute appendicitis” (14.5%).

### 3.2. Quality of Life & Anxiety

**Figure 1:**
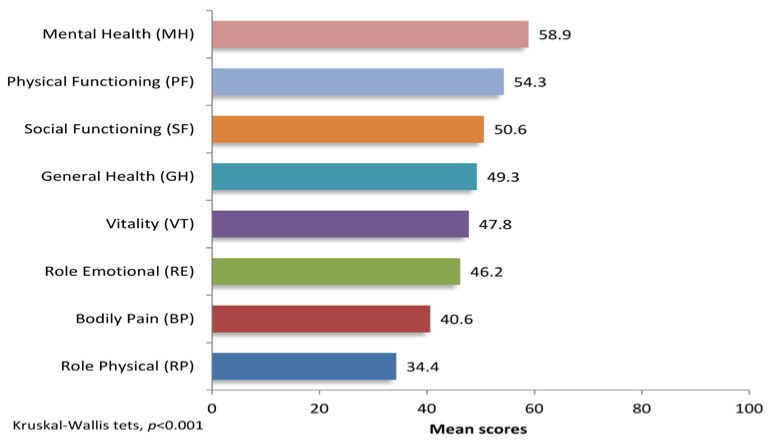
Hierarchical comparison of scores of the eight components of the SF-36 QoL scale for the 83 study participants.

**Figure 2:**
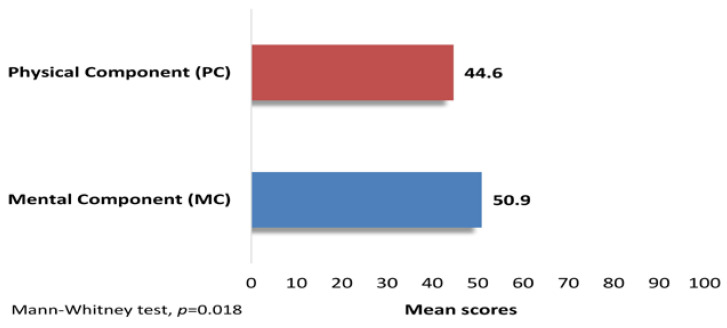
Comparison of scores of the two main components of the SF-36 QoL scale for the 83 study participants.

Table 2 presents the SF-36 QoL scores of the 83 participants. Moderate to high mean scores (>50.0) are recorded for Physical Functioning (54.3 ± 29.7), Social Functioning (50.6 ± 19.7) and Mental Health (58.9 ± 18.8), while in the main QoL components the Physical Component was just 44.9 ± 20.4 and the Mental Component was 50.9 ± 15.6. Similarly, moderate to high mean QoL scores were recorded for the Physical Component (34.9%) and the Mental Component (50.6%). Cronbach’s α for all components ranged from 0.680 to 0.900, indicating poor to excellent internal consistency. Τhe STAI scores demonstrated a moderate to high mean Anxiety score (45.5 ± 8.9), while 68.7% had a high State Anxiety score (>40.0). Cronbach’s α for the STAI was 0.807 or excellent. However, the hierarchical comparison of the eight components (Figure 1) showed that Mental Health presented the highest mean score, with Role Physical scoring lowest (58.9 versus 34.3, *p* < 0.001). Furthermore, the Mental Component scored significantly higher than the Physical Component (Figure 2) (50.9 versus 44.9, *p* = 0.029).

### 3.3. Characteristics, QoL & Anxiety

Table 3 compared the mean scores of the two main components of the SF-36 with regard to anxiety. Patients with a high state anxiety score had significantly lower mean Mental Component scores compared to patients with low state anxiety (47.3 versus 58.7, *p* = 0.002), and borderline non-significant Physical Component scores (42.0 versus 50.4, *p* > 0.05).

However, in addition to the previous rough estimates, Table 4 presents the multiple logistic regression correlation and evaluation of the OR of the higher Physical and Mental Components of QoL, with reference to the patients’ characteristics and state anxiety. For each added year of age, the odds of a moderate to high Physical Component fall significantly (OR = 0.95, *p* = 0.012). Similarly, married or partnered patients have significantly higher odds of a moderate to high Mental Component (OR = 6.59, *p* = 0.006), whereas those with high state anxiety have significantly lower odds of a moderate to high Mental Component (OR = 0.16, *p* = 0.005). In practical terms, then, it suggests that younger patients have better QoL in the Physical Component, while better QoL in the Mental Component is associated with marriage/partnering or lower state anxiety.

## 4. Discussion

The aim of the present study was to investigate the quality of life of patients suffering from disorders of the GI tract who were treated in a surgical clinic, and to evaluate associated factors. In brief, the following findings emerged: (a) moderate to high mean QoL scores, (b) the Mental Component had a significantly higher mean score than the Physical Component, (c) patients had moderate to high mean scores for state anxiety, with two-thirds presenting high anxiety, and (d) suggest that younger patients have better QoL in the Physical Component., while better QoL in the Mental Component is associated with marriage/partnering and lower state anxiety.

Several studies have investigated functional gastrointestinal disorders across all age groups, the anxiety they cause and the impact they have on patients’ quality of life. The initial assessment of researchers globally mainly concerns the number and frequency of these diseases, their nature, symptoms and impact on overall physical and mental health and quality of life [3,14,15,16,17]. Sperber and colleagues (2021) conducted a multinational study in 33 countries to investigate the prevalence of factors associated with FGIDs [3]. Of the 73,076 participants in the study, 40.3% suffered from at least one FGID, while FGIDS were more prevalent among women and were associated with lower quality of life and more frequent doctor visits.

In the context of the component characteristics of FGIDs, Hartono and colleagues (2012) attempted to investigate the prevalence and severity of anxiety and depression in 248 patients in Malaysia with functional dyspepsia (FD), nonerosive reflux disease (NERD) and IBS [18]. Using the Hospital Anxiety and Depression Scale (HADS), they estimated that increased anxiety is more common in patients with IBS, affecting 67.7% of these patients. The present study, although the patients were hospitalized with different causes of admission, found roughly the same frequency of increased anxiety, 68.7%.

Similarly, in a study of 44 outpatients with IBS of the Gastroenterology Department of the Clinical Hospital Centre Rijeka, with a mean age of 45.3 years, Pletikosić Tončić & Tkalčić (2017) assessed the relationship between the Pictorial Representation of Illness and Self-Measure (PRISM) and measures of QoL and anxiety in order to evaluate its use in identifying patients with severe symptoms and/or serious QoL impairment [19]. The ultimate aim was to identify who would benefit from psychological interventions. Overall, the assessment of anxiety via STAI showed lower mean scores (35.27 ± 12.47) than those of the patients in the present study (45.5 ± 8.9). In the SF-36 QoL assessment, Pletikosić Tončić & Tkalčić (2017) found higher mean scores in both the Physical (75.72 ± 13.92) and the Mental Component (66.92 ± 19.36) than those of the patients in the present study (50.9 and 44.9 respectively) [19].

In another study, Söderquist and colleagues (2020) attempted to investigate and characterise GI symptoms in relation to depressive symptoms and anxiety in young adult psychiatric patients compared to healthy controls [20]. They found that patients with and without psychotropic medication reported significantly more GI symptoms than controls, while physical and mental anxiety were the main predictors of the Gastrointestinal Symptom Rating Scale for Irritable Bowel Syndrome (GSRS-IBS) score. Correspondingly, a study of 306 IBS patients in Göteborg, Sweden showed that anxiety is a key factor affecting the severity of GI symptoms and QoL [21]. Overall, then, both the bibliography and the present study reveal the impact or at least the direct relationship between anxiety and quality of life in these patients. Youth is a factor that needs to be taken into account, as the present study found that younger patients had better QoL regarding the Physical Component, while marriage/partnering increased QoL regarding the Mental Component. Although the age of appearance of each disease or disorder was not included in our investigation, it may be reflected in the actual age of the patients.

Nevertheless, the issue of the quality of life “paradox” in patients with GI symptoms has been raised. In presenting their theory, van Tilburg & Murphy (2015) firstly stress the impact of the disease symptoms on patient’s lives physically, emotionally, occupationally, socially and cognitively [16]. The term “quality of life” is developed in research and clinical practice to determine the impact of the disease and evaluate the efficacy of care and treatment. The authors believe that despite the issues and their symptoms, patients often report good QoL: for example, children with FGIDs such as IBS, functional dyspepsia, functional abdominal pain and chronic constipation paradoxically demonstrate lower QoL than patients with organic disease (Crohn’s, ulcerative colitis and gastroesophagal reflux disease).

In addition to the findings of various studies including the present one, the international bibliography contains references to ways of treating the symptoms of GI issues that go beyond conventional medicine, to provide patients with relief and improve their QoL. These methods mainly aim to reduce patient anxiety, as a basic counterproductive factor in symptom severity. In a randomized open trial, Kanchibhotla and colleagues (2021) explored the benefits of a novel meditation technique called the Vaishvanara Agni meditation (VAM) [22]. 54 patients practiced VAM for 50 days, focusing particularly on the navel region and the digestive system. Over the course of the 50-day intervention (at day 0, 24 and 50), significant improvements were seen in patients’ QoL, especially their symptoms, physical strength and the psychological domain. However, other interventions to treat isolated disorders and symptoms involved physical exercise [23,24], maintaining normal body weight through appropriate diet [25], and limiting unhealthy behaviors in general [26].

### Limitations

The main weakness of the present study concerns the evaluation of factors that may determine the severity of the symptoms of various diseases of the gastrointestinal system. The persistence of the disease and the prolonged symptomatology may affect quality of life, not only negatively but also giving rise to the paradox described in the previous section. Moreover, the conditions of the COVID-19 pandemic led to various limitations on research outside clinics like that in the present study, the most important being the small sample of patients treated with regard to the types of disease for which they were admitted. Their low frequency did not permit comparative evaluation or the use of a control sample, which would have directly differentiated inpatients or even outpatients with various disorders. However, the present work, as a purely observational cross-sectional study, unprecedented in a clinic of this type, provided correlations that will form a basis of reference for future studies.

## 5. Conclusions

The aim of the present study was to investigate the quality of life of patients suffering from disorders of the GI tract who were treated in a surgical clinic, and to evaluate associated factors. It found moderate to high mean QoL scores, with the Mental Component scoring significantly higher than the Physical Component, moderate to high mean scores for state anxiety, and a correlation between better QoL in the Physical Component and lower patient ages, while better QoL in the Mental Component was associated with marriage/partnering and lower state anxiety. Action is necessary on a clinical or individual level, guided by health professionals, to promote appropriate dietary choices and interventions for the avoidance of behaviors that could harm or endanger patients’ health [25,26].

## Figures and Tables

**Table 1:** Descriptive characteristics of the 83 study participants.

		n	%
Gender	men/women	42/41	50.6/49.4
Age, years	18–49	35	42.2
	50–69	32	38.5
	70–93	16	19.3
Marital status	Unmarried, Divorced, Widowed	25	30.1
	Married, Partnered	58	69.9
Children	yes/no	69/14	83.1/16.9
Nationality	Greek/other	78/5	94.0/6.0
Place of residence	Rural	18	21.7
	Urban	65	78.3
Education	Primary school	18	21.7
	Lower Secondary school	16	19.3
	Higher Secondary school	14	16.9
	Higher education (University, Technical College)	18	21.7
	MA, MSc, PhD	17	20.4
Monthly income, €	<500	24	28.9
	500–999	23	27.7
	1000+	36	43.4

**Table 2:** SF-36 QoL scale components and STAI State Anxiety scores of the 83 study participants.

	Mean	SD	Median	Min.	Max.	Cronbach’s α
**SF-36** **QoL** **Components** ^a^						
Physical Functioning (PF)	54.3	29.7	50.0	0.0	100.0	0.900
Role Physical (RP)	34.3	41.6	0.0	0.0	100.0	0.897
Bodily Pain (BP)	40.6	24.3	40.0	0.0	80.0	0.791
General Health (GH)	49.3	18.0	50.0	0.0	100.0	0.697
Vitality (VT)	47.8	20.7	50.0	0.0	95.0	0.836
Social Functioning (SF)	50.6	19.7	50.0	12.5	100.0	0.680
Role Emotional (RE)	46.2	43.2	33.3	0.0	100.0	0.711
Mental Health (MH)	58.9	18.8	60.0	12.0	100.0	0.810
Physical Component (PC)	44.6	20.4	42.5	0.0	95.0	0.863
moderate/high (>50.0)	*n* = 29 or 34.9%					
Mental Component (MC)	50.9	15.6	50.2	19.3	86.3	0.787
moderate/high (>50.0)	*n* = 42 or 50.6%					
**Anxiety**						
State Anxiety (STAI) ^b^	45.4	8.9	47.0	26	66	0.807
low (up to 40)	*n* = 26 or 31.3%					
high (>40)	*n* = 57 or 68.7%					

^a^ Higher score (→100) indicates better QoL. ^b^ Higher score (→80) indicates higher anxiety.

**Table 3:** Comparison of the two main SF-36 QoL scale components of the 83 study participants for low and high state anxiety.

*QoL:*	State Anxiety				Δ-Difference	*p*-Value
	*Low (* *up to 40)*		*High (>40)*			
	Mean	SD	Mean	SD		
**Physical Component** ^a^	50.4	19.8	42.0	20.3	8.4	0.083
**Mental Component** ^a^	58.7	13.8	47.3	15.1	11.4	0.002

^a^ Higher score (→100) indicates better QoL. Mann-Whitney test.

**Table 4:** Multiple logistic regression analysis of higher scores for Physical and Mental Components of QoL with patients’ characteristics and state anxiety.

	SF-36 QoL (Moderate/High versus Low)					
	Physical Component			Mental Component		
	OR	95%CI	*p*-Value	OR	95%CI	*p*-Value
**Gender** (women versus men)	0.86	0.28–2.70	0.799	0.61	0.21, 1.78	0.362
**Age** (for each year of change)	0.95	0.91–0.99	0.012	0.96	0.93, 1.00	0.057
**Family status** (Married or Partnered versus Unmarried, Divorced, Widowed)	3.97	0.91–17.3	0.067	6.59	1.72, 25.3	0.006
**Children** (yes versus no)	1.48	0.27–8.01	0.650	1.38	0.25, 7.46	0.711
**Education** (for each level of change)	1.02	0.67–1.56	0.913	0.83	0.55, 1.27	0.394
**Place of residence** (Urban versus Rural)	1.53	0.36–6.63	0.567	1.39	0.38, 5.08	0.619
**Monthly income** (for each level of change, e.g., <€500, €500–1000 or >€1000)	1.44	0.69–3.00	0.336	0.99	0.48, 2.07	0.987
**State Anxiety** (high versus low)	0.56	0.17–1.85	0.341	0.16	0.05, 0.57	0.005
*pseudo R*^2^ *_Negelkerke_*	0.265			0.268		

OR, odds ratio; 95%CI, 95% confidence intervals.

## Data Availability

Datasets are available from the first author on reasonable request.

## References

[1] Greenwood-Van Meerveld B., Johnson A.C., Grundy D. (2017). Gastrointestinal Physiology and Function. Handbook of Experimental Pharmacology.

[2] Black C.J., Drossman D.A., Talley N.J., Ruddy J., Ford A.C. (2020). Functional gastrointestinal disorders: Advances in understanding and management. Lancet.

[3] Sperber A.D., Bangdiwala S.I., Drossman D.A., Ghoshal U.C., Simren M., Tack J., Whitehead W.E., Dumitrascu D.L., Fang X., Fukudo S. (2021). Worldwide Prevalence and Burden of Functional Gastrointestinal Disorders, Results of Rome Foundation Global Study. Gastroenterology.

[4] Drossman D.A. (2016). Functional Gastrointestinal Disorders: History, Pathophysiology, Clinical Features and Rome IV. Gastroenterology.

[5] Tack J., Stanghellini V., Mearin F., Yiannakou Y., Layer P., Coffin B., Simren M., Mackinnon J., Wiseman G., Marciniak A. (2019). Economic burden of moderate to severe irritable bowel syndrome with constipation in six European countries. BMC Gastroenterol..

[6] Wong R.K., Drossman D.A. (2010). Quality of life measures in irritable bowel syndrome. Expert Rev. Gastroenterol. Hepatol..

[7] Creed F., Ratcliffe J., Fernandez L., Tomenson B., Palmer S., Rigby C., Guthrie E., Read N., Thompson D. (2001). Health-related quality of life and health care costs in severe, refractory irritable bowel syndrome. Ann. Intern. Med..

[8] Kim Y.K., Shin C. (2018). The Microbiota-Gut-Brain Axis in Neuropsychiatric Disorders: Pathophysiological Mechanisms and Novel Treatments. Curr. Neuropharmacol..

[9] O’Mahony S.M., Clarke G., Dinan T.G., Cryan J.F., Greenwood-Van Meerveld B. (2017). Irritable Bowel Syndrome and Stress-Related Psychiatric Co-morbidities: Focus on Early Life Stress. Gastrointestinal Pharmacology.

[10] Anagnostopoulos F., Niakas D., Pappa E. (2005). Construct validation of the Greek SF-36 Health Survey. Qual. Life Res..

[11] Rikos N., Flouri M., Pandermaraki E., Spokos E., Linardakis M. (2022). Health-related quality of life of patients with rheumatic diseases in the Southern Aegean region, Greece. Arch. Hell. Med..

[12] Fountoulakis K.N., Papadopoulou M., Kleanthous S., Papadopoulou A., Bizeli V., Nimatoudis I., Iacovides A., Kaprinis G.S. (2006). Reliability and psychometric properties of the Greek translation of the State-Trait Anxiety Inventory form Y: Preliminary data. Ann. Gen. Psychiatry.

[13] Emons W.H., Habibović M., Pedersen S.S. (2019). Prevalence of anxiety in patients with an implantable cardioverter defibrillator: Measurement equivalence of the HADS-A and the STAI-S. Qual. Life Res..

[14] Choi M.G., Jung H.K. (2011). Health related quality of life in functional gastrointestinal disorders in Asia. J. Neurogastroenterol. Motil..

[15] Ross E.J., Vivier H., Cassisi J.E., Dvorak R.D. (2020). Gastrointestinal health: An investigation of mediating effects on mood and quality of life. Health Psychol. Open.

[16] Van Tilburg M.A., Murphy T.B. (2015). Quality of life paradox in gastrointestinal disorders. J. Pediatr..

[17] Wu J.C. (2012). Psychological Co-morbidity in Functional Gastrointestinal Disorders: Epidemiology, Mechanisms and Management. J. Neurogastroenterol. Motil..

[18] Hartono J.L., Mahadeva S., Goh K.L. (2012). Anxiety and depression in various functional gastrointestinal disorders: Do differences exist?. J. Dig. Dis..

[19] Pletikosić Tončić S., Tkalčić M. (2017). A Measure of Suffering in relation to Anxiety and Quality of Life in IBS Patients: Preliminary Results. BioMed Res. Int..

[20] Söderquist F., Syk M., Just D., Kurbalija Novicic Z., Rasmusson A.J., Hellström P.M., Ramklint M., Cunningham J.L. (2020). A cross-sectional study of gastrointestinal symptoms, depressive symptoms and trait anxiety in young adults. BMC Psychiatry.

[21] Jerndal P., Ringström G., Agerforz P., Karpefors M., Akkermans L.M., Bayati A., Simrén M. (2010). Gastrointestinal-specific anxiety: An important factor for severity of GI symptoms and quality of life in IBS. Neurogastroenterol. Motil..

[22] Kanchibhotla D., Sharma P., Subramanian S. (2021). Improvement in Gastrointestinal Quality of Life Index (GIQLI) following meditation: An open-trial pilot study in India. J. Ayurveda. Integr. Med..

[23] Martin D. (2011). Physical activity benefits and risks on the gastrointestinal system. South. Med. J..

[24] Sadeghian M., Sadeghi O., Hassanzadeh Keshteli A., Daghaghzadeh H., Esmaillzadeh A., Adibi P. (2018). Physical activity in relation to irritable bowel syndrome among Iranian adults. PLoS ONE.

[25] Axelrod C.H., Saps M. (2020). Global Dietary Patterns and Functional Gastrointestinal Disorders. Children.

[26] Saneei P., Esmaillzadeh A., Keshteli A.H., Roohafza H.R., Afshar H., Feizi A., Adibi P. (2021). Combined Healthy Lifestyle Is Inversely Associated with Upper Gastrointestinal Disorders among Iranian Adults. Dig. Dis..

